# Experimental Research on New Developed Titanium Alloys for Biomedical Applications

**DOI:** 10.3390/bioengineering9110686

**Published:** 2022-11-12

**Authors:** Cristina Jimenez-Marcos, Julia Claudia Mirza-Rosca, Madalina Simona Baltatu, Petrica Vizureanu

**Affiliations:** 1 Mechanical Engineering Department, Las Palmas de Gran Canaria University, 35017 Tafira, Spain; 2 Department of Technologies and Equipment for Materials Processing, Faculty of Materials Science and Engineering, Gheorghe Asachi Technical University of Iaşi, 700050 Iasi, Romania

**Keywords:** titanium alloys, Ti20Mo7Zr, Ti20Mo7Zr0.5Si, microstructure, corrosion behavior, mechanical properties

## Abstract

The mechanical properties and electrochemical behavior of two new titanium alloys, Ti20Mo7Zr and Ti20Mo7Zr0.5Si, are investigated in this paper. The alloys have been manufactured by vacuum arc remelting (VAR) technique and studied to determine their microstructure, corrosion behavior, and mechanical properties. Metallographic observations and quantitative microanalysis by optical microscopy, scanning electron microscopy SEM, and energy dispersive X-rays spectroscopy EDX were performed. Data about the three-point bending test and microhardness are presented. For electrochemical properties, three different environments were used: Ringer solution at 25 °C, Ringer solution at 40 °C simulating fever condition, and 3.5% NaCl solution. Metallographic investigation revealed the biphasic and dendritic structure of both samples when the procedures were performed. Electrochemical testing in body simulation fluid, fever conditions, and saline medium showed that the lower the proportion of silicon in the samples, the higher the corrosion resistance. The formation of a titanium oxide layer on the surface of both samples was noticed using quantitative EDX analysis. The three-point bending test for the two samples revealed that the presence of silicon decreases the modulus of elasticity; the surface of the samples displayed soft and hard phases in the microhardness test. Electrochemical impedance spectroscopy (EIS) measurements were carried out at different potentials, and the obtained spectra exhibit a two-time constant system, attesting double-layer passive film on the samples.

## 1. Introduction

Nowadays, progress in medicine and materials development has made possible the study, design, and application of numerous biomaterials covering a wide range of medical fields covering orthopedics, drug administration, dentistry, skin tissue engineering, and cardiovascular systems, among many others, for the benefit of the human being [1,2]. In this way, people’s quality of life and longevity can be improved. However, as human life expectancy rises, so does the elderly population, which is more prone to chronic musculoskeletal diseases such as osteoarthritis. It is believed that up to 15% of the population is affected by osteoarthritis [3].

For this reason, any biomaterial has to meet a number of requirements, the main one being biocompatibility with the human body, which is the ability of a material to fulfill its function in medical treatment by generating the most appropriate beneficial cellular or tissue response in that specific situation and optimizing the clinically relevant performance of that treatment [4,5]. In order to prevent implant loosening and to obtain a longer duration of service, which would save revision surgery, these biomaterials must have the following properties: high ductility, high fatigue and wear resistance, absence of cytotoxicity, and a combination of high strength and low Young’s modulus that is equivalent to human cortical bone and ranges from 10 to 30 GPa. Furthermore, the implant must integrate well with the nearby bone [2,5,6,7,8].

Biomedical implants based on polymers and ceramics show low mechanical strength and brittleness, which limits their applications for harsh working conditions [9]. Therefore, currently, within orthopedic surgery, it is necessary to develop prostheses made mainly of metallic materials.

Around 70–80% of implants are made of metallic biomaterials [7]. The most commonly used metals for medical applications are 316L stainless steel, CoCrMo alloys, and Ti and its alloys, such as Ti6Al4V and NiTi alloys [5,10]. The modulus of elasticity of metals is generally much higher than that of bone and prevents load transfer to the bone. The stress shielding effect can cause osteoporosis due to the excessive difference between the elastic modulus of metal and bone. In addition, there are few adequate metal candidates recommended for their use as long-term implants due to the toxic effect of the ions that they release in the adjacent tissues, their poor resistance to wear and corrosion, and, therefore, their low biocompatibility. Nevertheless, these issues can be solved by mixing the base metal with alloying components that do not exhibit any harmful impact on the body [2,5,9,11].

One of the most significant risks that can be caused by a metallic implant is corrosion, which is the deterioration of a material due to an electrochemical attack in its environment. Losses resulting from corrosion are estimated at hundreds of billions of dollars each year. This is in the order of 2–4% of the Gross National Product [12]. In order to prevent this phenomenon; a film is usually produced on the surface of the material, which makes it impermeable and protects it from the environment.

In general, titanium and its alloys have triumphed with respect to more traditional and are today widely used in aerospace, industrial, and biomedical applications, especially as tissue substitutes, such as dental implants, femoral heart valves, and fracture plates, due to the possibility of modifying their properties by changing the composition of the alloying elements, their biocompatibility with biological materials, their high corrosion resistance, high mechanical performance, low modulus and high thermal stability [2,3,8,12,13,14,15,16,17]. Due to titanium’s poor electrical conductivity, which aids in the material’s electrochemical oxidation by forming a highly stable passive oxide coating on its surface naturally, this material is biocompatible. The material is hence very resistant to corrosion [1,6,16,18].

There are three different kinds of titanium-based alloys: α, α + β, and β titanium. When heated above 882 °C, pure titanium changes from its alpha structure, known as the compact hexagonal structure (HCP), to its beta structure, body-centered cubic (BCC), as a result of an allotropic transition. Yet, the beta phase may often be kept at ambient temperature in this element’s alloys, resulting in a material that has both phases. Additionally, changes in alloy characteristics such as ductility, plasticity, or formability result from differences in the relative quantities of different phases. These metallographic transformations and the properties of titanium alloys can be improved by the addition of α-stabilizers (C, N, O, Al), which increase the allotropic transformation temperature, β-stabilizers (V, Nb, Mo, Ta, Fe, Mn, Cr, Co, W, Ni, Cu, Si), which decrease the allotropic transformation temperature and the α / β ratio is affected, leading to an increase in the amount of β phase and finally neutral stabilizers (Zr, Sn, Hf, Ge, Th) [2,19,20,21,22,23,24,25].

Ti6Al4V alloy is the most widely used of all titanium alloys for orthopedic applications, as it has excellent mechanical properties, corrosion resistance in biofluids due to the stable passive oxide layer formed on its surface, biocompatibility and its properties can be modified by heat treatment. However, it does not have good wear resistance, even with soft tissue friction. Furthermore, it is highly toxic, as it causes harmful tissue reactions caused by the release of vanadium ions, which is a toxic and carcinogenic element and can even lead to aseptic loosening of the prosthesis, and of aluminum ions which, in high concentrations, can cause certain long-term health disorders such as peripheral neuropathy, dementia, apathy or severe tremors, Alzheimer’s and Parkinson’s diseases and adverse tissue effects [15,26,27,28,29,30,31,32].

Taking all of this into account, four elements, Ti, Mo, Zr, and Si, were chosen for the construction of these new alloys because there is an important demand to develop new Ti alloys that have good mechanical properties and contain no toxic elements.

Ti-Mo alloys were investigated because molybdenum is a less poisonous element than common metals such as cobalt, nickel, and chromium, it is a β-stabilizing element, and some research suggests that adding molybdenum results in mechanical qualities resembling those of human bone. It also has low cost, high melting temperature, thermal stability, and corrosion resistance, being able to form a highly adherent stable oxide film (MoO_3_) that stops titanium corrosion [11,12,19,33,34,35,36].

Ti-Mo-Zr alloys were developed because zirconium is becoming a desirable alternative for the majority of medical applications. It is highly biocompatible with human tissues, has a low modulus of elasticity, and is very resistant to corrosion [19,25,37,38].

New Ti-Mo-Zr-Si alloys were recently fabricated when small quantities of Si were added to Ti-Mo-Zr because silicon is an element present in human bone and is deemed biocompatible due to its effects on corrosion resistance, creep resistance, ductility, and titanium strength at high temperatures. Similar to molybdenum, it is a β-stabilizing element that affects the decline in the elastic modulus [19,32,37,39].

Thus, two new TiMoZrxSi alloys have been synthesized using a vacuum arc remelting furnace (VAR), and their microstructure, corrosion behavior, quantitative microanalysis, modulus of elasticity, and hardness were all examined in this study. These methods included metallography, electrochemical, scanning electron microscopy, three-point bending, and microhardness tests.

## 2. Materials and Methods

### 2.1. Material Preparation

We have carried out the analysis of two different compositional alternatives of new alloys with Titanium, Molybdenum, Zirconium, and Silicon, with the purpose of performing different experiments in order to determine the effect produced by the addition of silicon in one of the samples and in this way, to obtain their properties. The chemical composition of the studied alloys is TiMoZr (73% Ti, 20% Mo, 7% Zr) and TiMoZr0.5Si (72.5% Ti, 20% Mo, 7% Zr, 0.5% Si). The raw materials used were high-purity elements such as Ti (99% purity), Mo (99% purity), and Zr (99% purity) supplied by Alfa Aesar by Thermo Fisher Scientific. The production of these alloys was carried out at the Faculty of Materials Science and Engineering from Ghe, Asachi Technical University of Iasi, Romania, using a vacuum arc remelting furnace (VAR) where a consumable electrode was fused in a vacuum at a monitored rate using the heat produced by an electric arc between the electrode and the ingot. To achieve an adequate uniformity of the alloys, these were centrifuged and remelted six times (three times in each part) in an inert atmosphere of argon and ultimately solidified in an ingot. Arc melting was chosen for developing the new TiMoZrxSi alloys because the method produces ingots of the highest purity. Part of the ingots was sent to Las Palmas de Gran Canaria University for their preparation and testing.

First of all, the surfaces of the two samples were prepared by embedding them by adding a 4:1 ratio of epoxy resin in molds. It is to say, for every 4 drops of resin, a drop of catalyst was added. The samples were then cut longitudinally with a thickness of 1 to 1.5 mm using the Buehler IsoMet 4000 precision saw (Buehler, Lake Bluff, IL, USA). Furthermore, vertical cuts of about 0.5 mm thickness were performed employing the cutting device. Then, grinding and polishing were performed in two stages using the Struers TegraPol-11 polishing machine (Struers ApS, Ballerup, Denmark ): using progressive grit silicon carbide papers from 280 to 1200 and the final polishing by applying 0.1 microns of alpha alumina suspension to polish the surfaces to a mirror finish. The experimental steps were in accordance with ASTM E3-11(2017) for the preparation of metallography samples.

**Figure 1:**
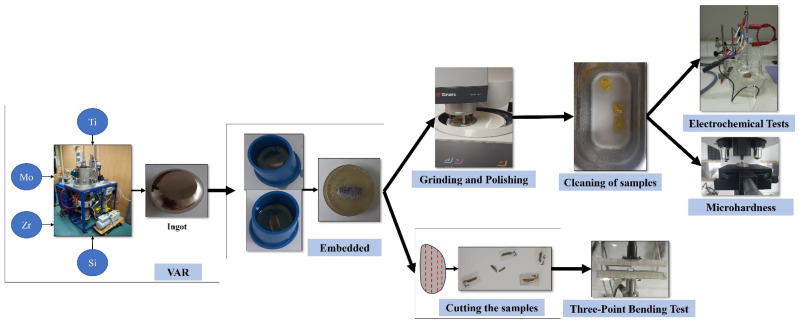
Schematic representation of the material preparation.

Finally, the samples were immersed in a heated “Ultrasons-HD” ultrasonic apparatus from J.P. Selecta (JPS, Barcelona, Spain) for 10 minutes to remove all traces of dirt and impurities (see Figure 1).

The titanium alloys under study, as mentioned above, have a passive titanium oxide layer that protects them from the medium, and this layer has different properties from those of the base metal. Thus, to obtain a correct hardness measurement, first, a lower load is applied to make the indenter penetrate to a certain depth, and then progressively higher loads are applied to obtain the hardness values of the base metal.

### 2.2. Metallography

For this test, images of the sample surfaces were taken using the Axio Vert.A1 MAT ZEISS (Zeiss, Jena, Germany) metallographic optical microscope, magnifying 10 and 20 times their actual size. Each sample was immersed in a Kroll etching reagent composed of H_2_O, HNO_3_, and HF for 15 seconds, and images of the etched surface were taken.

### 2.3. Electrochemical Tests

In order to carry out these tests, a sample (working electrode) in Ringer’s solution is introduced in the electrochemical cell together with two electrodes: the saturated calomel electrode (SCE) is used as the reference electrode, and the platinum electrode acts as the counter electrode. The Ringer Grifols solution (from Grifols Laboratories, Barcelona, Spain) had the following contents in mmol/L: Na+ 129.9; K+ 5.4; Ca2+ 1.8; Cl− 111.7; and C_3_H_5_O_3_ 27.2.

Five techniques were applied using the BioLogic Essential SP-150 potentiostat (Bio-Logic Science Instruments SAS, Seyssinet-Pariset, France): Corrosion Potential, Electrochemical Impedance Spectroscopy in Ringer’s solution, in saline medium, and in fever state, Corrosion Rate, Corrosimetry, and Pitting Potential.

#### 2.3.1. Corrosion Potential

In order to measure the corrosion potential, the “Ecorr vs. time” technique was applied for 24 h, with potential values of ±10 V and with a potential recording every 300 s or every time varying by 200 mV. The data obtained were plotted as a graph of potential vs. time, which may remain constant with respect to time or show a trend toward passivation or corrosion.

#### 2.3.2. Electrochemical Impedance Spectroscopy (EIS)

For the impedance measurement, “Potential Electrochemical Impedance Spectroscopy” was selected, and the surface value and the measurement duration of 5 minutes were entered. This measurement was performed 7 times for each sample, at ± 300 mV vs. Ecorr in Ringer’s solution, with maximum and minimum potential values of ±10 V. These data were represented by Bode and Nyquist diagrams and equivalent circuits.

This same technique, with the same data, was applied in the simulation of saline and fever-state media. In the saline medium, however, the saline solution was first created using 35 g of NaCl in 1 L of solution, and the sample was immersed for 24 h. On the other hand, in the case of fever states, the electrochemical cell was immersed in a Grant Instruments Y14 thermostatic bath (Grant Instruments, Cambridgeshire, England), applying heat (40 °C) for 24 h. After the measurements were taken, they were plotted on bode diagrams.

#### 2.3.3. Corrosion Rate

In order to perform these measurements, the “Linear Polarization” technique was selected, and the sample surface area value and the test duration of 20 minutes were entered to stabilize its potential. The maximum and minimum potential values were ±10 V, with the potential scanning presenting a 0.167 mV/s time-variation relationship from −0.025 to 0.025 V versus open circuit potential (Eoc), with data recording every 0.5 s and intensity throughout the potential scanning at 100%. These linear polarization curves were then plotted, and EC-Lab’s “Tafel Fit” analysis was applied to find the corrosion rate values for each sample.

#### 2.3.4. Corrosimetry

The measurement of the Rp was performed by applying the “Corrosimetry” technique within the software and filling in the configuration window the parameters such as surface area, equivalent weight, and density of the samples with the duration time of each cycle to be performed being a total of 10 cycles for each sample, in addition to parameters previously obtained by means of linear polarization (βa and βc). The maximum and minimum potential values were ±10 V, and the potential scan showed a time-varying ratio of 0.167 mV/s from −0.025 to 0.025 V versus Eoc, with data recorded every 1 s and intensity throughout the potential scan at 100%. Next, polarization resistance data were plotted versus time.

#### 2.3.5. Pitting Potential

In order to carry out the test, the “Cyclic Potentiodynamic Polarization” technique was selected where the applied potential scan was −0.7 V and 2 V with respect to the reference electrode and with a reverse scan of up to −0.5 V with a data recording every 0.5 s and of the intensity every time 200 mA or every 1 s varies. The maximum and minimum values of the potential were ±10 V. After performing the test for each sample, the graphs obtained were curves as a function of current intensity (mA) and potential (V).

### 2.4. Scanning Electron Microscopy (SEM)

In order to realize this test, the samples were prepared and placed inside the Zeiss Sigma 300 VP (Zeiss, Jena, Germany) microscope using a sample holder, and a vacuum was applied. Then, using SmartSEM, an image of the surface of the sample was taken at 1000× magnification and for 20kV, and, using SmartEDX, the quantitative microanalysis of the sample was carried out, obtaining the percentages of the elements and the spectrum.

### 2.5. Three-Point Bending Test

The three-point bending method was carried out using the Bose ElectroForce^®^ 3100 machine (Bose Corporation, Framingham, MA, USA), which complies with ISO 7438:2020 and can withstand up to 20 N of applied force. In this case, each specimen of rectangular cross-section, with a length varying from approximately 13 to 12 mm, was placed at the extremities of the bottom shank of the testing device, allowing enough space between the supports, which ranged from 7.80 to 10.63 mm, depending on the length of the specimens. Then, a vertical load with a linear velocity of 3 mm/s was applied at the center point of the specimen until the specimen exceeded its yield strength or split. In order to determine the modulus of elasticity, the obtained values of the applied load versus the displacement of the specimens were plotted, and their slope was obtained.

### 2.6. Microhardness

According to ISO 14577-1:2015, 10 indentations were made for each applied load of each sample, in this case, 5 gf, 25 gf, and 50 gf using the Affri DM8 B hardness tester. When very small loads are applied, there is a good chance that the mark will be found in only one phase so that the hardness of that phase can be evaluated; as the load increases, the mark may cover parts of several phases, giving an idea of the total hardness of the material. Then, using the Aries software, the lengths of the diagonals were measured, and the Vickers microhardness values were automatically calculated. These hardness values were plotted against the number of indentations performed.

## 3. Results and Discussion

### 3.1. Metallography

Metallography is the study of the structural or constitutive characteristics of metals or alloys in order to relate them to their physical and mechanical properties. In this way, the spatial structure of the phases and compounds that make up a metallic material may be demonstrated in this method, and the impurities, fiber orientation, and potential mechanical flaws in the samples can also be determined [40].

**Figure 2:**
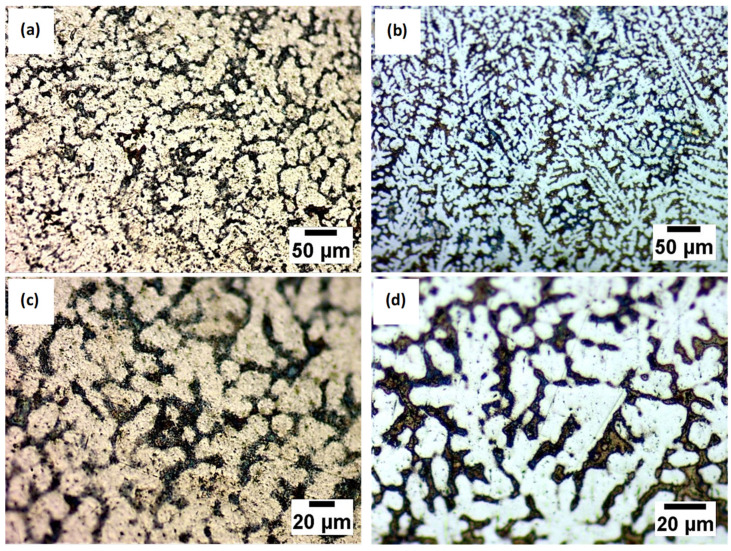
Microstructure by optical microscopy after chemical etching for different magnifications of the Ti20Mo7Zr (**a**,**c**) and Ti20Mo7Zr0.5Si (**b**,**d**) sample.

After the electrochemical etching representative, metallographic images of the analyzed alloys are presented in Figure 2.

While cpTi has an HCP structure, or an α-phase, at ambient temperature, there is a BCC structure, called β-phase, at temperatures over 883 °C. With the inclusion of β-stabilizers such as Mo and Si, the β-phase is stable at temperatures below 883 °C; in contrast, Zr is regarded as a neutral element since it almost has no impact on the α/β phases [2].

It can be observed that both analyzed samples, after being attacked, contain a biphasic and dendritic structure, distinguishable for both magnifications. Furthermore, the size of dendrites decreased by almost 25% with the Si addition while the interdendritic zone increased.

### 3.2. Electrochemical Tests

#### 3.2.1. Corrosion Potential

The corrosion potential is the point at which the cathodic current density becomes anodic when a metal is introduced into the solution, although, according to the Mixed Potential Theory developed by Wagner and Traud [41], the oxidation and reduction reactions in corrosion occur at the same rate at the surface of the metal.

The evolution of corrosion potential with time is used as a quantitative criterion for corrosion behavior but remains insufficient for a complete analysis.

**Figure 3:**
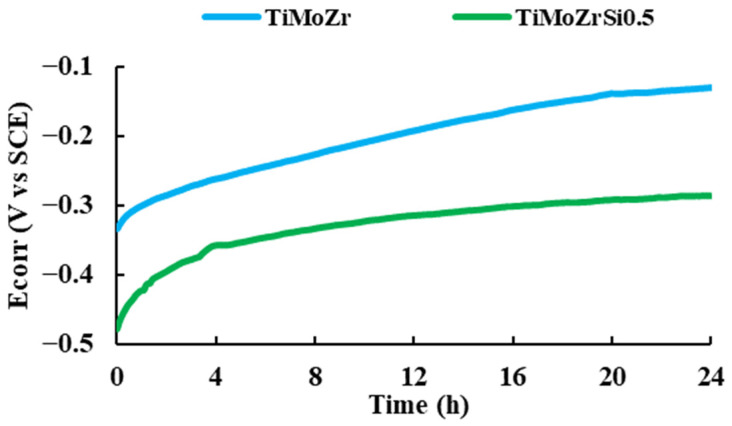
Corrosion potential vs. time for 24 h of immersion in Ringer solution.

We noted that the corrosion potential of both samples increased during the immersion time, indicating the passivation of the samples in the Ringer solution. The curves did not exhibit potential drops associated with surface activation during the exposure, suggesting that the passive film is thermodynamically resistant in these conditions (see Figure 3).

The corrosion potential of Ti20Mo7Zr0.5Si (−0.286 V) is more negative than that of Ti20Mo7Zr (−0.150 V) and of cpTi (between −0.1 V and −0.15 V), suggesting that the characteristics of passive film changed with Si addition [42].

#### 3.2.2. Electrochemical Impedance Spectroscopy (EIS)

Electrochemical Impedance Spectroscopy (EIS) allows the characterization of material properties and electrochemical systems by applying a variable frequency sine electric potential perturbation to the study and recording the current response within the electrochemical cell. Electrochemical impedance spectroscopy (EIS) is a high-performance technique used for the examination of interfacial characteristics associated with processes that occur on the surface of metallic alloys. With respect to other electrochemical techniques, EIS provides various benefits as it is a stationary state technique, which involves the measurement of small signals and is capable of probing, in our case, from 100 mHz to 100 KHz.

##### Bode and Nyquist diagrams in Ringer’s solution

**Figure 4:**
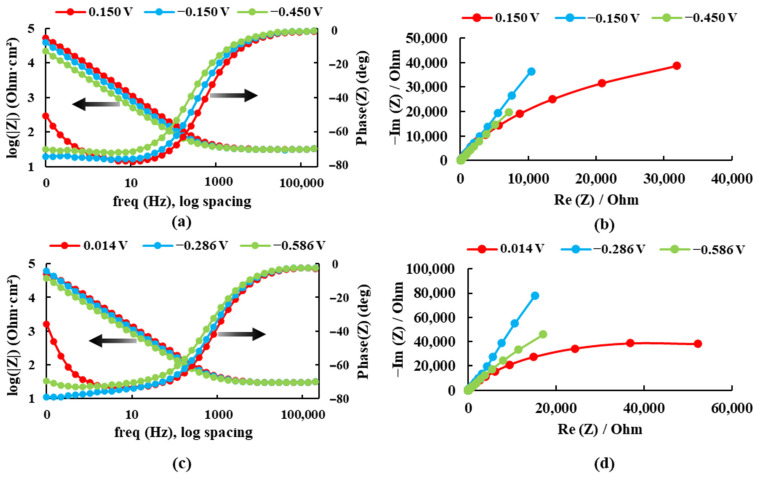
Bode and Nyquist diagrams for: (**a**,**b**) Ti20Mo7Zr and (**c**,**d**) Ti20Mo7Zr0.5Si in Ringer’s solution.

The electrochemical impedance spectroscopy results are presented in Bode plots (see Figure 4) for both alloys recorded at three different potentials in the Ringer solution. The maximum impedance value and the maximum phase angle for each case are presented in Table 1.

For the sample Ti20Mo7Zr, when analyzing Figure 4a and Table 1, the impedance and phase angle values tend to increase as the applied potential becomes more positive. The process occurs in a single stage (time constant) as the curve tends to decrease. Whereas, for the Ti20MoZr sample, both Figure 4b and Table 1 show an increase in the impedance and phase angle values as the applied potential value increases, apart from the 0.014 V potential curves, where the values decrease, and it is observed that, at 0.1 Hz, the Bode Phase curve presents two-time constants. Therefore, the maximum corrosion resistance value was obtained by the Ti20Mo7Zr0.5Si sample.

##### Equivalent Circuits

**Figure 5:**
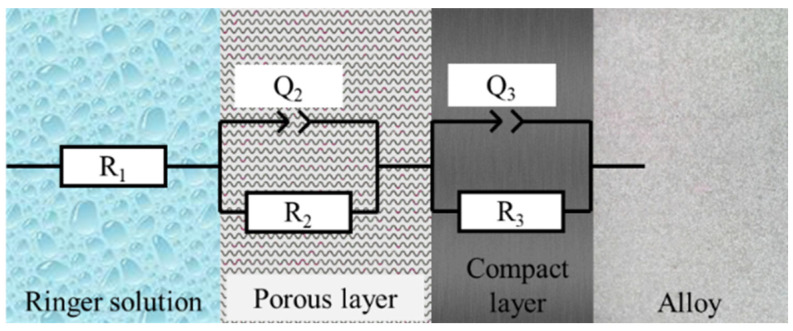
Equivalent circuit R(QR)(QR).

For all the EIS spectra of cpTi, a capacitive behavior can be observed that was fitted by the compact passive film model [42] in Figure 5, the circuit that has been adapted to the results obtained by means of the Bode diagrams in Ringer’s solution is R(QR)(QR). This circuit indicates that the samples present a resistance to dissolution and two passive layers (porous and compact) until the alloy is reached.

(1) $$Z\left(f\right)=R_{1}+\frac{R_{2}}{R_{2}Q_{2}{\left(j2\pi f\right)}^{n_{2}}+1}+\frac{R_{3}}{R_{3}Q_{3}{\left(j2\pi f\right)}^{n_{3}}+1}$$

Equation (1) indicates the impedance value for the equivalent circuit R(QR)(QR). According to Equation (1), the value of the impedance depends on the following parameters:

- R_1_: Resistance of the dissolution.
- R_2_: Resistance of the porous layer.
- R_3_: Resistance of the compact layer.
- Q_2_ and Q_3_: Constant phase element.
- f: Frequency.
- n_2_ and n_3_: Parameter indicating whether the constant phase element simulates a capacitor (n = 1), a semi-infinite Warburg impedance (n = 0.5), or a resistor (n = 0).

##### Sample Ti20Mo7Zr

**Figure 6:**
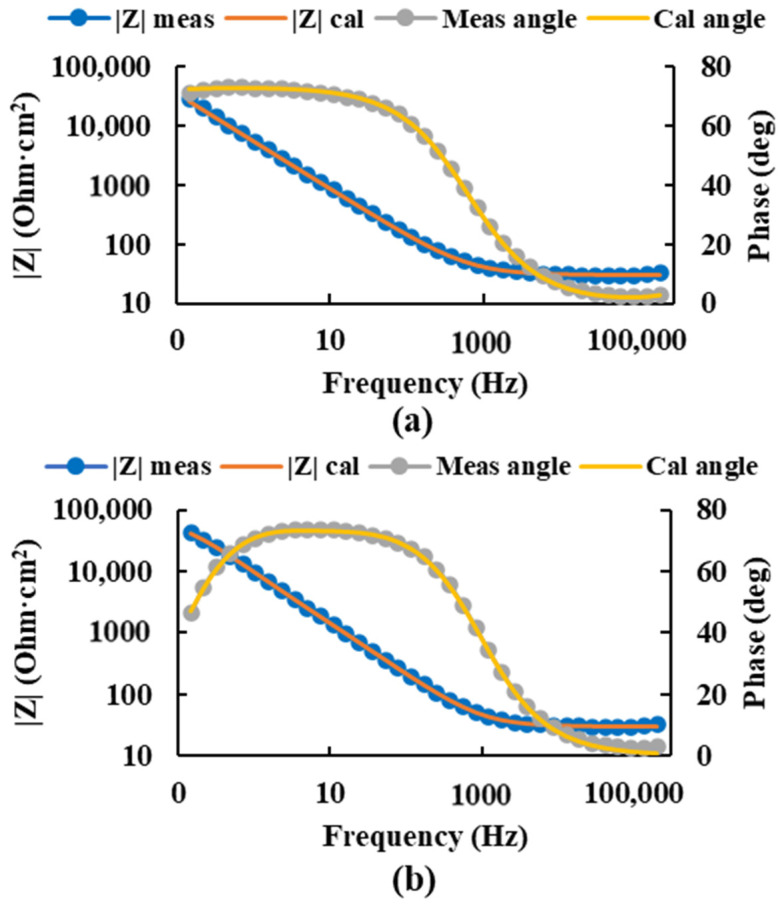
Bode diagram of the measured and calculated impedance and angle of the Ti20Mo7Zr sample at potentials −0.450 V (**a**) and 0.150 V (**b**) using the equivalent circuit R(QR)(QR).

After the selection of the equivalent circuit, the values of the circuit resistances and the constant phase elements (Table 2) were obtained, and the values of the impedance and phase angle measured and calculated by this circuit were plotted for the most negative and the most positive potential (Figure 6), thus obtaining the value of the corrosion resistance of the passive layers higher for the most positive potential.

##### Sample Ti20Mo7Zr0.5Si

**Figure 7:**
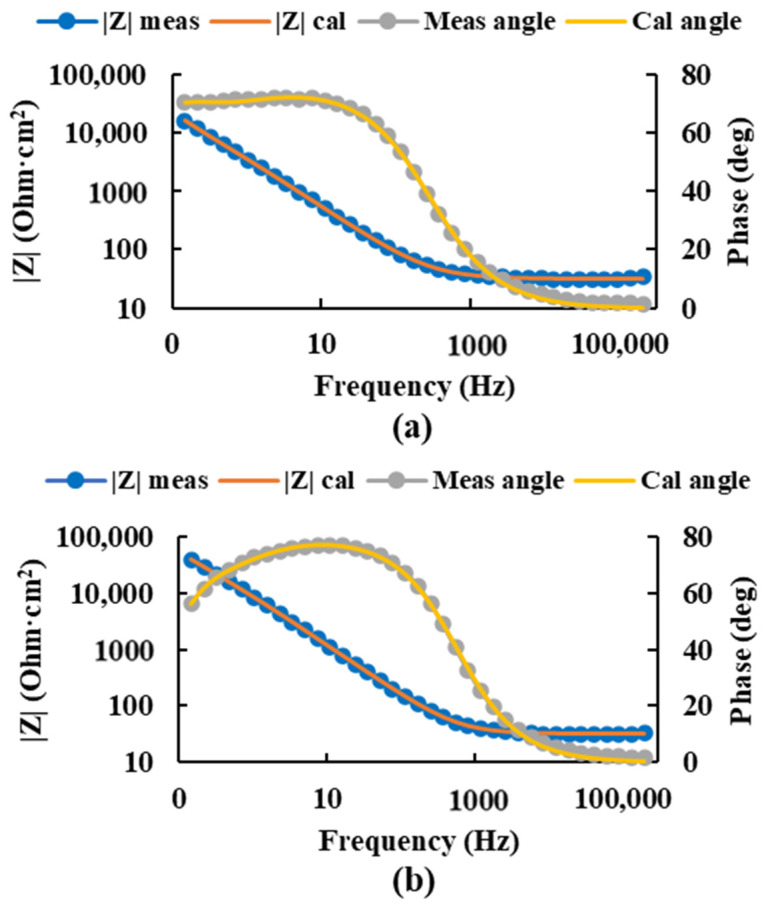
Bode diagram of the measured and calculated impedance and angle of the Ti20Mo7Zr0.5Si sample at potentials −0.586 V (**a**) and 0.014 V (**b**) using the equivalent circuit R(QR)(QR).

For the Ti20Mo7Zr0.5Si sample, the measured impedance and phase angle values were plotted as calculated by the circuit using Figure 7, and the values of the circuit resistances and constant phase elements were obtained (Table 3). In this case, the values of the corrosion resistance of the porous layers were higher for the more positive potential.

In this case, higher corrosion resistance was obtained in the compact layer for the most negative potential. However, in the porous layer, it was higher for the most positive potential.

##### Simulation of Saline Environments

**Figure 8:**
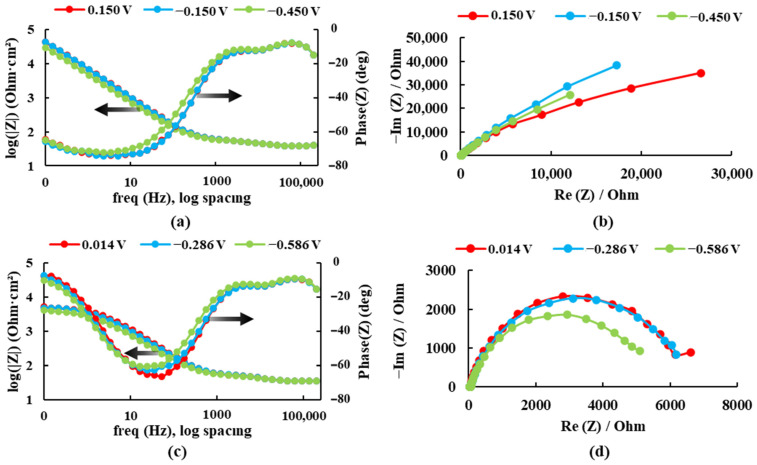
Bode and Nyquist diagrams for: (**a**,**b**) Ti20Mo7Zr and (**c**,**d**) Ti20Mo7Zr0.5Si in saline environments.

In view of the Bode diagram curves in Figure 8 and the results in Table 4, it is observed that the values of the logarithm of the impedance and phase angle tend to increase with the application of a more positive potential and, therefore, the corrosion resistance increases, which is maximum for the Ti20Mo7Zr0. 5Si sample. The dissolution process of the Ti20Mo7Zr sample occurs in two stages, while that of the Ti20Mo7Zr0.5Si sample occurs in three stages, as the phase angle curve grows, decreases, grows, decreases, and grows again.

##### Simulation of Fever States (40 °C)

**Figure 9:**
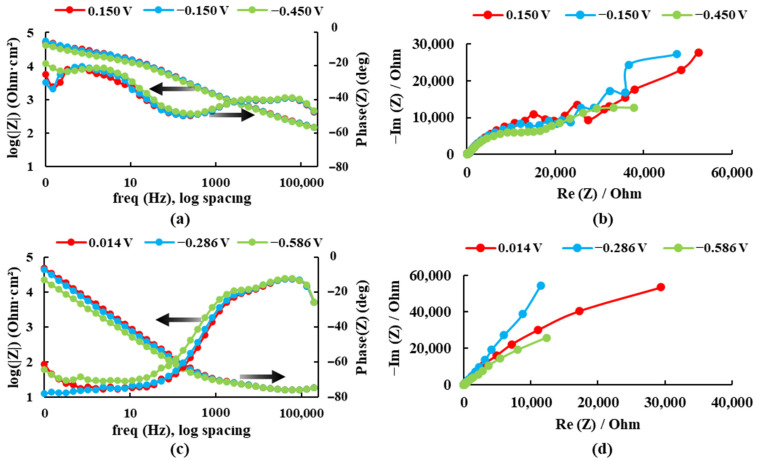
Bode and Nyquist diagrams for: (**a**,**b**) Ti20Mo7Zr and (**c**,**d**) Ti20Mo7Zr0.5Si in Ringer’s solution at 40 °C.

As in the salt medium simulation, it can be seen in Figure 9 and Table 5 that the impedance and phase angle values tend to increase as the applied potential becomes more positive. The dissolution process of the Ti20Mo7Zr sample occurs in three stages as the phase angle curve grows, decreases, grows, and decreases again. However, in the Ti20Mo7Zr0.5Si sample, only two-time constants are observed. In this case, the highest corrosion resistance value was presented by Ti20Mo7Zr.

#### 3.2.3. Corrosion Rate

The corrosion rate provides information about the impact of an environment on material and can be found by means of linear polarization curves, which are represented by potential and intensity.

**Figure 10:**
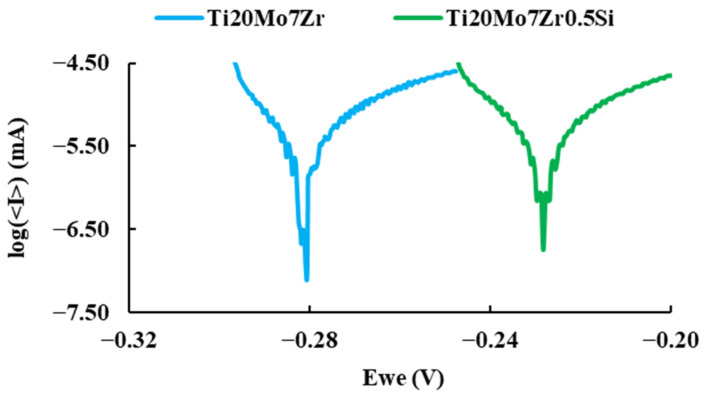
Comparison of the polarization curves of the samples.

The linear polarization test carried out to determine the corrosion rate of the alloys, plotted on a semi-logarithmic scale of current values, is presented in Figure 10.

It can be observed more anodic corrosion potential values for Ti20Mo7Zr0.5Si than those of Ti20Mo7Zr. The corrosion current (i_corr_) is indicative of the degree of oxidation of the alloy, and it can be observed that it is bigger for the alloy with Si addition. The Tafel slopes (β_a_ and β_c_) were determined by analyzing the curve plotted in a range of ±250 mV vs. open circuit potential (OCP). An alloy with a tendency to passivate will have a value of βa greater than βc, whereas an alloy that tends to corrode will have an anodic slope smaller than the cathodic slope. In our case (see Table 6), both alloys tend to passivate.

Table 6 shows the parameters of the Tafel line and the corrosion rate (CR) of the tested samples, which is expressed in thousandths of an inch of annual penetration (mpy) according to the following equation:

$$\mathrm{CR}=\frac{I_{\mathrm{corr}}\times \;K\;\times \;\mathrm{EW}}{d\;\times \;A}$$

where:

I_corr_ is corrosion current (in A).

K is the constant that defines the units of the corrosion rate (1.288 × 10^5^ miliinches/A-cm-year).

EW is the equivalent weight (in g/cm^3^).

A is the sample area (in cm^2^).

In this case, a maximum corrosion rate of 3.50 × 10^−3^ mpy (Ti20Mo7Zr0.5Si) and a minimum corrosion rate of 2.63 × 10^−3^ mpy (Ti20Mo7Zr) were obtained, while cpTi in the same conditions has a higher corrosion rate of 4.8 × 10^−3^ mpy [42].

#### 3.2.4. Corrosimetry

Corrosimetry is designed to follow the evolution of standard corrosion values (R_p_, E_corr_, I_corr_) as a function of time. The polarization resistance (R_p_) is defined as the slope of the potential-current density curve at the free corrosion potential and is inversely proportional to the uniform corrosion rate.

**Figure 11:**
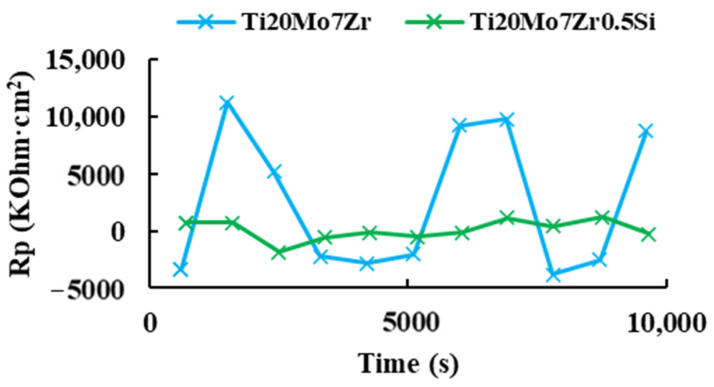
Polarization resistance versus time of Ti20Mo7Zr and Ti20Mo7Zr0.5Si.

After obtaining the corrosion rate, Figure 11 shows the variation of the polarization resistance with respect to the 3 hours’ time for the two study samples.

For both samples, it was not possible to appreciate a behavior of decrease or increase in the polarization resistance with respect to the time, being in the case of Ti20Mo7Zr0.5Si, the obtained values of the polarization resistance were very dispersed during the duration of the test. Then, it could be verified in the EC-Lab software manual that this is a technique that requires application over long periods of time (months). Therefore, it was decided to apply the “R_p_ Fit” analysis of the EC-Lab software to the linear polarization plots of the corrosion rate, where the X-axis shows the potential Vs. the reference electrode, while the Y-axis shows the current intensity.

The data obtained for the polarization resistance are shown in Table 7. As the value of the polarization resistance (R_p_) is bigger, the alloy is more resistant to corrosion. For highly corrosion-resistant materials, the R_p_ may even reach 10^6^ Ω·cm^2^, so both alloys are very resistant to corrosion.

#### 3.2.5. Pitting Potential

**Figure 12:**
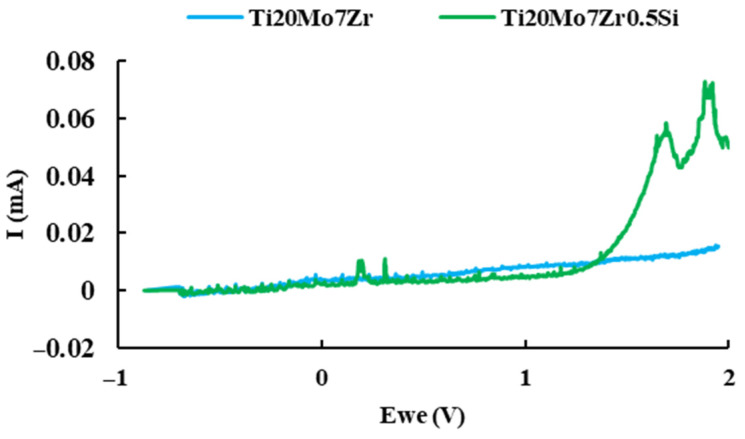
Current versus potential curve of Ti20Mo7Zr and Ti20Mo7Zr0.5Si samples.

Pitting is one of the most insidious forms of localized corrosion in which small holes appear on the surface of the material since the passive layer on the surfaces of many metals breaks down in the presence of ionic concentrations such as chlorides and bromides. The pitting corrosion behavior of titanium alloy samples in Ringer’s solution was examined by cyclic potentiodynamic polarization at room temperature, and the plots of intensity versus time were plotted in Figure 12.

The Ti20Mo7Zr sample reaches the maximum 2 V set in the test parameters without corroding. The same happens with Ti20Mo7Zr0.5Si, although, in this case, when the sample reaches 2 V, the intensity values are higher.

### 3.3. Scanning Electron Microscopy (SEM)

Scanning electron microscopy is a topographical, structural, and compositional analysis technique widely used in the study of semiconductors or nanoparticles, among others.

**Figure 13:**
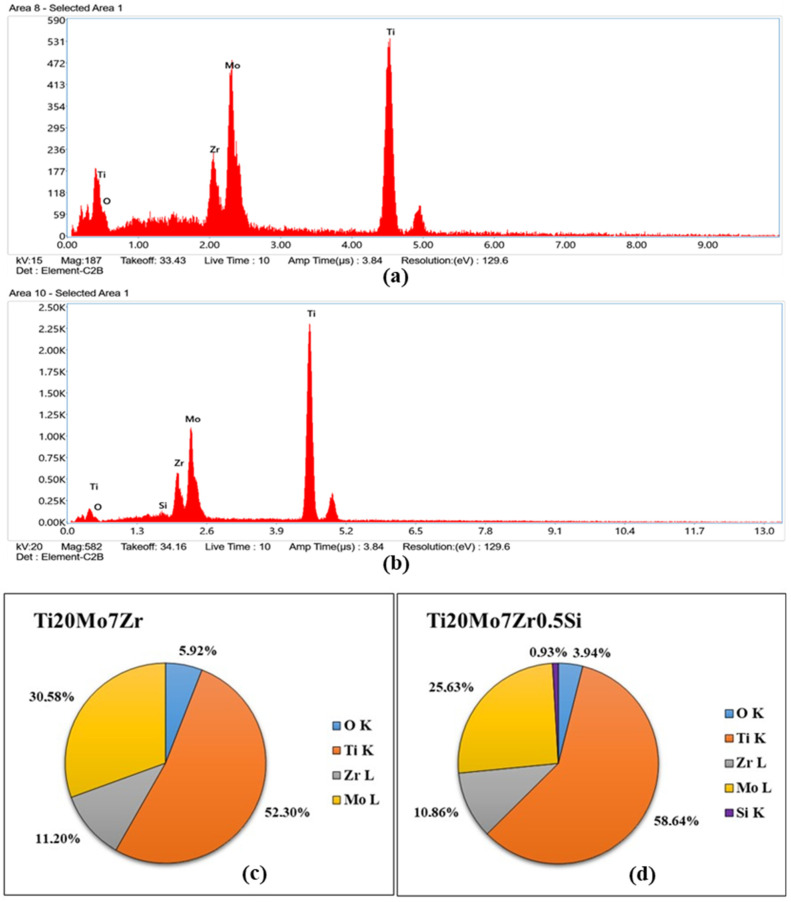
EDX spectra for Ti20Mo7Zr (**a**) and Ti20Mo7Zr0.5Si (**b**) samples and quantification (**c**,**d**).

Energy dispersive X-rays spectroscopy EDX results for the investigated area of each sample after the corrosion process are presented in Figure 13.

EDX spectra show the energy dispersive obtained for both samples, which indicate the presence of the constituent elements in addition to oxygen (see Figure 13c,d), probably due to the formation of the titanium oxide layer and the corrosion processes carried out previously. On the other hand, with respect to the quantitative values provided, by selecting only a small area of the part of the sample surface at 1000 magnification by SEM, the percentages differ with respect to those produced by the manufacturer due to the fact that on the alloy’s surface there are dendrites and interdendritic areas and the area selected for the microanalysis could have been found in one of the different phases presented by the sample. It can be observed that there is one area rich in Mo, Zr, and Si and another area rich in Titanium.

### 3.4. Three-Point Bending Test

After determining the values of the modulus of elasticity for each specimen, it was decided to calculate the mean of the samples tested, taking into account their mean deviation, which corresponds to the arithmetic mean of the absolute values of the deviations from the mean.

In Table 8, the average value of the modulus of elasticity of the tested specimens of the Ti20Mo7Zr sample was 86.85 ± 10 GPa, while, in the case of sample Ti20Mo7Zr0.5Si, it was obtained at 49.33 ± 12 GPa, which is lower than the other specimen and close to Young’s modulus of human bone.

In turn, these results obtained are in similar ranges to those found in previous research for the TiMoZrTa alloy (52–69 GPa). However, if zirconium is eliminated in the alloy of this study (TiMoSi), lower values of the modulus of elasticity (20–43 GPa) close to that of human bone (7–30 GPa) are obtained [19]. On the other hand, when compared to more common dental and commercially used alloys such as CoCrMo (210–253 GPa), stainless steel (190–210 GPa), Ti6Al4V (119 GPa), C. P. Ti (105 GPa), it can be stated that the new alloy, TiMoZrSi, has a much lower modulus of elasticity.

### 3.5. Microhardness

Generically, this test consists of applying a vertical load on the surface of the samples using the pyramidal tetrahedral indenter of the hardness tester, which has an angle between edges of 136°, until the indentation is created and, following the Vickers method, the load applied, and the area left in the indentation are related.

For the analysis of the results obtained in this technique, according to the equivalent circuit obtained in the electrochemical tests, it must be taken into account that these alloys have different layers: the porous passive layer, the compact passive layer, and the base metal. Therefore, if a very small load is applied, the indenter will only reach the porous layer, but with increasing loads, the hardness of the base metal can be reached.

**Figure 14:**
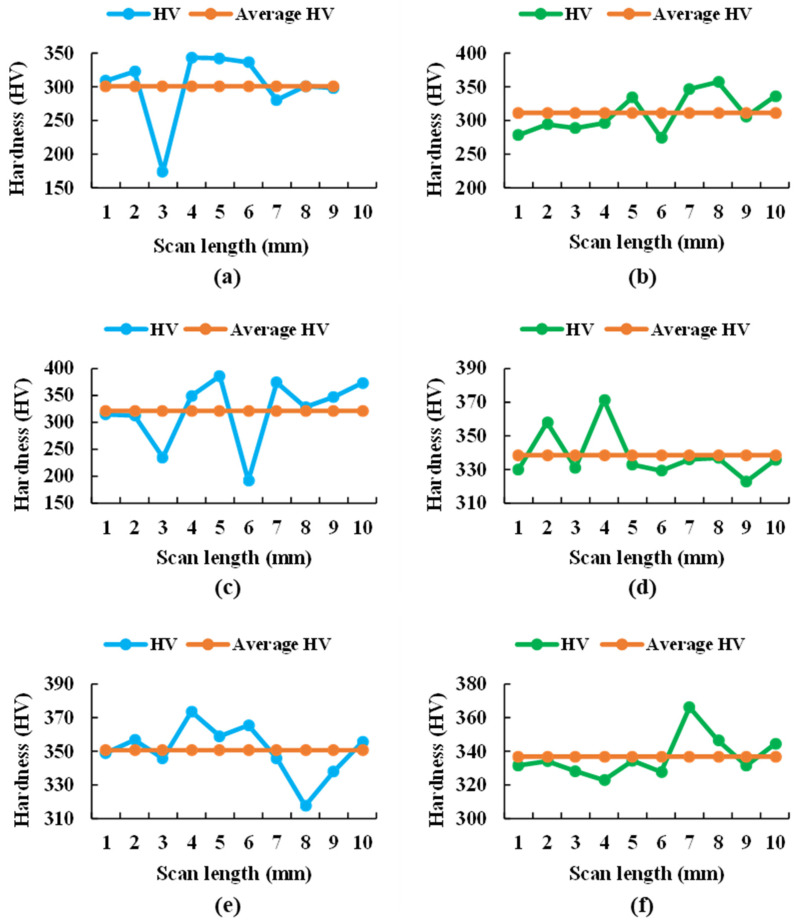
Microhardness values of each indentation for the 5 (**a**), 25 (**c**), and 50 g (**e**) loading of the Ti20Mo7Zr sample and for the 5 (**b**), 25 (**d**), and 50 g (**f**) loading of the Ti20Mo7Zr0.5Si sample.

For both samples, the graphs in Figure 14 show widely dispersed Vickers hardness values of each indentation for each sample at each applied load (5, 25, and 50 gf), attributed to the different hardness values of the α and β phases, as well as the crystalline orientation within the material. Therefore, the surface of the samples had soft and hard zones, as shown in Table 9.

Thus, the maximum values of the hard phase obtained during this test with respect to the Vickers microhardness occurred in the Ti20Mo7Zr0.5Si sample, while in the soft zone of Ti20Mo7Zr0.5Si, they are slightly higher. This phenomenon is due to the previous forming of the alloy. Moreover, the maximum hardness values obtained in this study were lower than those of common alloys such as Ti6Al4V (541 HV) and CoCrMo (155-601 HV) [19] but higher than for cpTitanium [43].

## 4. Conclusions

This research evaluated and compared the corrosion behavior and mechanical properties of two titanium alloys. By means of microstructural analysis, corrosion potential curves, EIS, corrosion rate, pitting potential, corrosimetry, SEM, three-point bending test, and microhardness, the following conclusions have been reached:

- The surfaces of the attacked samples have similar dendritic biphasic structures. The size of the dendrites decreases with the addition of silicon. On the surface of the alloys, there is one area rich in Mo, Zr, and Si and another area rich in Titanium. The presence of a passive oxide layer on the surface of each sample was detected by EDX results, with a porous aspect in contact with the electrolyte and a compact structure in contact with the alloy.
- The potential of both samples increased during the immersion time with no drops in potential values, which demonstrated that the passive film formed on the surface is thermodynamically resistant in these conditions. The polarization resistance (Rp) is very big, so both alloys are very resistant to corrosion.
- The low corrosion rates and low corrosion currents testify to the good performance of the studied samples in the analyzed environment, Ringer solution, saline solution, and fever simulation.
- The modulus of elasticity and hardness values obtained for both new alloys were lower than those of many commercial alloys. Moreover, the addition of silicon decreases the modulus of elasticity by almost 45% compared to the alloy without silicon, thus approaching the modulus of elasticity of bone.

In view of the results obtained, the new alloys showed good mechanical properties (hardness and modulus of elasticity) and high corrosion resistance in comparison with commercial alloys.

## Figures and Tables

**Table 1:** Results obtained in the Bode diagrams for the Ti20Mo7Zr and Ti20Mo7Zr0.5Si samples.

Alloys	Ti20Mo7Zr			Ti20Mo7Zr0.5Si		
Potential [V]	−0.450	−0.150	0.150	−0.586	−0.286	0.014
Max. Impedance [Ω]	21,380	38,905	51,286	38,019	60,256	50,119
Max. phase angle [°]	72	76	77	73	79	74

**Table 2:** Equivalent circuit R(QR)(QR) of sample Ti20Mo7Zr.

Potential	R_1_ (Ω)	Y_01_ (S × Sec^n^)	n_1_	R_2_ (Ω)	Y_02_ (S × Sec^n^)	n_2_	R_3_ (Ω)	χ^2^
**−0.450 V**	31.55	1.48 × 10^−4^	0.76	3.41 × 10^3^	7.25 × 10^−5^	0.95	1.18 × 10^5^	4.73 × 10^−4^
**0.150 V**	31.47	2.78 × 10^−5^	0.98	8.00 × 10^4^	5.53 × 10^−5^	0.82	7.72 × 10^3^	4.45 × 10^−4^

**Table 3:** Equivalent circuit R(QR)(QR) of sample Ti20Mo7Zr0.5Si.

Potential	R_1_ (Ω)	Y_01_ (S × Sec^n^)	n_1_	R_2_ (Ω)	Y_02_ (S × Sec^n^)	n_2_	R_3_ (Ω)	χ^2^
**−0.586 V**	18.2	6.87 × 10^−9^	0.99	1.16 × 10^1^	3.98 × 10^−5^	0.81	2.01 × 10^6^	5.19 × 10^−4^
**0.014 V**	29.6	1.97 × 10^−5^	0.89	7.36 × 10^4^	1.52 × 10^−4^	0.75	4.78 × 10^2^	5.49 × 10^−4^

**Table 4:** Results obtained in Bode diagrams for Ti20Mo7Zr and Ti20Mo7Zr0.5Si in saline environments.

Alloys	Ti20Mo7Zr			Ti20Mo7Zr0.5Si		
Potential [V]	−0.450	−0.150	0.150	−0.586	−0.286	0.014
Max. Impedance [Ω]	29,512	43,652	45,709	3981	4786	5129
Max. phase angle [ °]	72	74	73	60	63	66

**Table 5:** Results obtained in Bode diagrams for Ti20Mo7Zr and Ti20Mo7Zr0.5Si a 40 °C.

Alloys	Ti20Mo7Zr			Ti20Mo7Zr0.5Si		
Potential [V]	−0.450	−0.150	0.150	−0.586	−0.286	0.014
Max. Impedance [Ω]	40,738	56,234	61,660	22,387	42,658	47,863
Max. phase angle [ °]	48	50	50	71	78	76

**Table 6:** Corrosion parameters entered and obtained for all samples tested.

Parameters	Ti20Mo7Zr	Ti20Mo7Zr0.5Si
**E_corr_ (mV vs. Ref)**	−228.76	−282.06
**I_corr_ (nA/cm^2^)**	1.94	2.58
**β_c_ (mV/dec)**	12.8	12.9
**β_a_ (mV/dec)**	19.2	17.3
**Equivalent weight (g/eq)**	60.52	60.42
**Density (g/cm^3^)**	5.75	5.77
**Surface (cm^2^)**	1.03	0.78
**Corrosion rate (mpy)**	2.63 × 10^−3^	3.50 × 10^−3^

**Table 7:** Polarization resistance of Ti20Mo7Zr and Ti20Mo7Zr0.5Si applying “R_p_ Fit.”

Parameters	Ti20Mo7Zr	Ti20Mo7Zr0.5Si
**Rp (Ohm·cm^2^)**	836,389	1,080,000
**E_corr_ (mV)**	−226.32	−233.88
**I_corr_ (μA/cm^2^)**	3.99 × 10^−3^	2.97 × 10^−3^

**Table 8:** Modulus of elasticity values obtained from the tested specimen of Ti20Mo7Zr and Ti20Mo7Zr0.5Si.

Sample	E Average (GPa)
Ti20Mo7Zr	86.85 ± 10.16
Ti20Mo7Zr0.5Si	49.33 ± 12.00

**Table 9:** Microhardness values of applied loads of soft and hard phase Ti20Mo7Zr and Ti20Mo7Zr0.5Si.

	Ti20Mo7Zr		Ti20Mo7Zr0.5Si	
Load (g)	Microhardness (HV)		Microhardness (HV)	
	Soft Phase	Hard Phase	Soft Phase	Hard Phase
**5**	174	343	275	357
**25**	192	386	323	371
**50**	318	374	323	366

## Data Availability

All data provided in the present manuscript are available to whom it may concern.
